# Impact of age and comorbidity burden on mortality and major complications in older adults undergoing orthopaedic surgery: an analysis using the Japanese diagnosis procedure combination database

**DOI:** 10.1186/1471-2474-14-173

**Published:** 2013-05-28

**Authors:** Hirotaka Chikuda, Hideo Yasunaga, Hiromasa Horiguchi, Katsushi Takeshita, Shurei Sugita, Shuji Taketomi, Kiyohide Fushimi, Sakae Tanaka

**Affiliations:** 1 Department of Orthopaedic Surgery, Faculty of Medicine, The University of Tokyo, 7-3-1 Hongo, Bunkyo-ku, Tokyo 113-8655, Japan; 2 Department of Health Management and Policy, Graduate School of Medicine, The University of Tokyo, 7-3-1 Hongo, Bunkyo-ku, Tokyo 113-8655, Japan; 3 Department of Health Care Informatics, Tokyo Medical and Dental University, 1-5-45 Yushima, Bunkyo-ku, Tokyo 113-8510, Japan

**Keywords:** Orhopaedic surgery, Spine surgery, Arthroplasty, Complication, Mortality, Database, Charlson comorbidity index, Elderly patients

## Abstract

**Background:**

The purpose of this study was to examine how complications in older adults undergoing orthopaedic surgery vary as a function of age, comorbidity, and type of surgical procedure.

**Methods:**

We abstracted data from the Japanese Diagnosis Procedure Combination database for all patients aged ≥ 50 who had undergone cervical laminoplasty, lumbar decompression, lumbar arthrodesis, or primary total knee arthroplasty (TKA) between July 1 and December 31 in the years 2007 to 2010. Outcome measures included all-cause in-hospital mortality and incidence of major complications. We analyzed the effects of age, sex, comorbidities, and type of surgical procedure on outcomes. Charlson comorbidity index was used to identify and summarize patients’ comorbid burden.

**Results:**

A total of 107,104 patients were identified who underwent cervical laminoplasty (16,020 patients), lumbar decompression (31,605), lumbar arthrodesis (18,419), or TKA (41,060). Of these, 17,339 (16.2%) were aged 80 years or older. Overall, in-hospital death occurred in 121 patients (0.11%) and 4,448 patients (4.2%) had at least one major complication. In-hospital mortality and complication rates increased with increasing age and comorbidity. A multivariate analysis showed mortality and major complications following surgery were associated with advanced age (aged ≥ 80 years; odds ratios 5.88 and 1.51), male gender, and a higher comorbidity burden (Charlson comorbidity index ≥ 3; odds ratio, 16.5 and 5.06). After adjustment for confounding factors, patients undergoing lumbar arthrodesis or cervical laminoplasty were at twice the risk of in-hospital mortality compared with patients undergoing TKA.

**Conclusions:**

Our data demonstrated that an increased comorbid burden as measured by Charlson comorbidity index has a greater impact on postoperative mortality and major complications than age in older adults undergoing orthopaedic surgery. After adjustment, mortality following lumbar arthrodesis or cervical laminoplasty was twice as high as that in TKA. Our findings suggest that an assessment of perioperative risks in elderly patients undergoing orthopaedic surgery should be stratified according to comorbidity burden and type of procedures, as well as by patient’s age.

## Background

Orthopaedic surgery for degenerative spine or limb joints is widely performed with the aim of improving patients’ quality of life. In the United States, about 400,000 spinal arthrodesis and 600,000 total knee arthroplasty (TKA) operations are completed annually [1,2]. Orthopaedic surgery for elderly patients, even octogenarians and nonagenarians, is becoming more common as the population ages. This trend is particularly evident in Japan, where 23% of the population are 65 years or older [3,4].

Despite the rapid increase of surgical treatment in elderly patients, the impact of advanced age on the risk arising from orthopaedic surgery is not fully understood. Although age has long been considered as a major risk factor for perioperative mortality and morbidity, chronological age is not the sole determinant of surgical risk. Risk factors vary considerably among individuals and depend on multiple variables including severity of disease, comorbid conditions, and type of surgical procedure. Further understanding of each factor’s contribution would allow surgeons to better evaluate perioperative risk to elderly patients. However, few large studies, in particular those using a national database, has examined this issue [1,5-10].

In this analysis of nationwide inpatient claim data, we investigated mortality and morbidity in older adults undergoing one of the following operations: cervical laminoplasty; lumbar decompression; lumbar arthrodesis; and primary TKA to examine how complications vary as a function of age, comorbid conditions, and type of surgical procedure.

## Methods

### Data source

The Diagnosis Procedure Combination (DPC) is a case-mix patient classification system launched in 2002 by the Ministry of Health, Labour and Welfare of Japan, and linked with a lump-sum payment system [11-14]. All 82 university teaching hospitals are obliged to use this system, but adoption by community hospitals is voluntary. The DPC Research Group surveys participating hospitals between July 1 and December 31 each year in collaboration with the Ministry of Health, Labour and Welfare. In 2010, 926 hospitals participated and provided data for 2.9 million patients, approximately 45% of all inpatient admissions to acute care hospitals in Japan. The database includes the International Classification of the Diseases 10th Revision (ICD-10) codes for primary and secondary diagnoses; comorbid conditions that existed at admission; and complications that occurred after admission. To optimize the accuracy of the recorded diagnoses, physicians in charge are obliged to record the diagnoses with reference to medical charts.

The Institutional Review Board at The University of Tokyo approved the study design and waived informed consent because the data is anonymous.

### Patients

We included all patients aged 50 years or older who had undergone one of the following operations from 2007 to 2010: cervical laminoplasty, lumbar decompression, lumbar arthrodesis, and primary TKA. These procedures were chosen because they are predominantly performed for degenerative conditions commonly seen in the elderly. Although these procedures might be conducted in an urgent manner, this study did not exclude emergency cases. We excluded total hip arthroplasty (THA) from our analysis because of demographic and etiologic differences; the majority of Japanese patients present with osteoarthritis secondary to dysplasia of the hip joint, which is not necessarily the case in other parts of the world.

The variables abstracted from the DPC database were: age, sex, comorbid conditions that existed at admission, surgical site, length of stay, and postoperative adverse outcomes. To evaluate the impact of multiple comorbidities, we used Charlson comorbidity index (CCI) base on Quan’s protocol [15,16], a weighted index that takes into account the number and the seriousness of comorbid conditions. In calculation of the CCI, patients’ comorbid conditions are classified into following 17 categories: myocardial infarction; congestive heart failure; peripheral vascular disease; cerebrovascular disease; dementia; chronic pulmonary disease; peptic ulcer diease; mild liver disease; diabetes without chronic complication; diabetes with chronic complication; hemiplesia or paraplegia; renal disease; any malignancy, including leukemia and lymphoma; moderate or severe liver disease; metastatic solid tumor; and AIDS/HIV. Each condition is assigned a score of 1, 2, 3, or 6, depending on the risk of dying associated with the condition. Scores are then summed to provide a total score of the CCI. In the present study, coded comorbidities in the DPC database were converted to designated points according to Quan’s protocol.

### Outcomes

Primary outcomes included in-hospital death or any of the following complications: surgical site infection (ICD10 code: T793, T814), sepsis (A40, A41), cardiac events (acute coronary events [I21-I24] or heart failure [I50]), respiratory complications (pneumonia [J12-J18], post procedural respiratory disorders [J95] or respiratory failure [J96]), pulmonary embolism (I26), stroke (cerebral infarction or hemorrhage [I60-I64]), acute renal failure [N17].

### Statistical analysis

We used analysis of variance or Kruskal-Wallis tests to compare continuous data; Chi-square tests to compare categorical data; Fisher’s exact test to compare in-hospital mortality and major complication rates between subgroups; logistic regression to analyze concurrent effects of factors on the occurrence of in-hospital deaths and postoperative complications, and a generalized estimating equation to adjust for the clustering of patients within hospitals. In the regressions, in-hospital deaths and postoperative complications were modeled as functions of age, sex, CCI, and surgical procedure [17]. The threshold for significance was < 0.05.

## Results

A total of 107,104 patients were identified (45,044 men and 62,060 women; mean ± SD age, 70.1 ± 10.7 years): cervical laminoplasty (16,020 patients); lumbar decompression (31,605); lumbar arthrodesis (18,419); or primary TKA (41,060). Of these, 17,339 (16.2%) were aged ≥ 80 years. Diabetes was the most common comorbid condition that existed at admission (found in 16.0% of the patients) followed by chronic pulmonary disease (2.6%), chronic renal failure (1.9%), malignancy (1.7%), and congestive heart failure (1.6%). Table 1 shows the characteristics of the patients according to surgical procedures.

**Table 1:** Characteristics of the study population according to surgical procedures

	**Overall**	**Cervical laminoplasty**	**Lumbar decompression**	**Lumbar arthrodesis**	**Total knee arthroplasty**	**p**
	**(n = 107,104)**	**(n = 16,020)**	**(n = 31,605)**	**(n = 18,419)**	**(n = 41,060)**	
**Age, years**; mean [SD]	70.1 [10.7]	66.2 [11.6]	68.9 [11.3]	65.7 [12.1]	74.6 [6.9]	< 0.001
≤ 64	25,927 (24.2)	6,534 (40.8)	8,898 (28.2)	6,976 (37.9)	3,519 (8.6)	
65–79	63,838 (59.0)	7,678 (47.9)	18,166 (57.5)	10,060 (54.6)	27,394 (68.0)	
≥ 80	17,339 (16.2)	1,808 (11.3)	4,541 (14.4)	1,383 (7.5)	9,607 (23.4)	
**Sex**						
Men	45,044 (42.1)	11,050 (69.0)	18,735 (59.3)	8,456 (45.9)	6,803 (16.6)	< 0.001
Women	62,060 (57.9)	4,970 (31.0)	12,870 (40.7)	9,963 (54.1)	34,257 (83.4)	
**Charlson comorbidity index**						
0	68,931 (64.4)	9,518 (59.4)	20,234(64.0)	11,971 (65.0)	27,208 (66.3)	< 0.001
1	24,068 (22.5)	3,481 (21.7)	7,060(22.3)	3,825 (20.8)	9,702 (23.6)	
2	9,656 (9.0)	1,885 (11.8)	2,928(9.3)	1,747 (9.5)	3,096 (7.5)	
≥ 3	4,449 (4.2)	1,136 (7.1)	1,383(4.4)	876 (4.8)	1,054 (2.6)	
**Postoperative length of stay,** day, median [IQR]	21 [15–30]	18 [14–26]	15 [12-21]	20 [15–28]	27 [21–36]	< 0.001

*SD* standard deviation, *IQR* interquartile range.

One-hundred and twenty-one patients died in-hospital following surgery (0.11%), and 4,448 patients (4.2%) experienced at least one major complication (Table 2). The most common major complication was surgical site infection (about 2% of patients) followed by cardiac events and respiratory complications. As expected, in-hospital mortality and complication rate increased with age and comorbid burden (Table 2). Patient receiving cervical laminoplasty showed the highest mortality (0.20%) and major complication rate (4.7%).

**Table 2:** Mortality and major complications following surgery according to age groups

	**Cervical laminoplasty**	**Lumbar decompression**	**Lumbar arthrodesis**	**Total knee arthroplasty**	**p**
	**(n = 16,020)**	**(n = 31,605)**	**(n =18,419)**	**(n = 41,060)**	
**Inhospital death, n (%)**	32 (0.20)	35 (0.11)	27 (0.15)	27 (0.066)	< 0.001
Age ≤ 64 years	5(0.077)	4 (0.045)	3 (0.043)	1 (0.028)	
65–79	17(0.22)	20 (0.11)	17 (0.17)	15 (0.055)	
≥ 80	10(0.55)	11 (0.24)	7 (0.51)	11 (0.11)	
**Postoperative complications, n (%)**					
Surgical site infection	320 (2.0)	511 (1.6)	362 (2.0)	636 (1.5)	< 0.001
Sepsis	25 (0.16)	53 (0.17)	53 (0.29)	61 (0.15)	0.002
Cardiac events	279 (1.7)	508 (1.6)	302 (1.6)	677 (1.6)	0.754
Respiratory complications	96 (0.60)	104 (0.33)	88 (0.48)	196 (0.48)	< 0.001
Pulmonary embolism	16 (0.10)	31 (0.10)	31 (0.17)	167 (0.41)	< 0.001
Stroke	71 (0.44)	74 (0.23)	42 (0.23)	92 (0.22)	< 0.001
Acute renal failure	1 (0.006)	8 (0.025)	7 (0.038)	15 (0.037)	0.227
**At least one complication, n (%)**	757 (4.7)	1,197 (3.8)	808 (4.4)	1,686 (4.1)	< 0.001
Age ≤ 64 years	264 (4.0)	254 (2.9)	248 (3.6)	106 (3.0)	
65–79	384 (5.0)	716 (3.9)	467 (4.6)	1,114 (4.1)	
≥ 80	109 (6.0)	227 (5.0)	93 (6.7)	466 (4.9)	

Mortality and major complication following surgery were associated with advanced age (aged ≥ 80 years; odds ratios [OR], 5.88 and 1.51 respectively), male gender, and increasing comorbidity (CCI ≥ 3; OR, 16.5, and 5.06 respectively) (Table 3). We note the risk of in-hospital death following lumbar arthrodesis or cervical laminoplasty was twice that in TKA.

**Table 3:** Adjusted risk of adverse outcomes after surgery

	**Inhospital death**			**Postoperative complications**				
	**OR**	**95% CI**	**p**	**OR**	**95% CI**			**p**
**Age**								
≤ 64	Reference			Reference				
65–79	2.58	1.36 – 4.89	0.004	1.22	1.09 – 1.36			0.001
≥ 80	5.88	2.93 – 11.8	<0.001	1.51	1.31 – 1.75			< 0.001
**Sex**								
men	Reference			Reference				
women	0.60	0.39 – 0.92	0.018	0.88	0.82 – 0.95			0.001
**Charlson comorbidity index**								
0	Reference			Reference				
1	1.60	0.89 – 2.87	0.116	2.42	2.17 – 2.71			< 0.001
2	6.58	3.99 – 10.8	<0.001	3.42	2.92 – 4.01			< 0.001
≥ 3	16.50	10 – 27.2	<0.001	5.06	4.2 – 6.1			< 0.001
**Surgical procedure**								
Total knee arthroplasty	Reference			Reference				
Cervical laminoplasty	2.15	1.23 – 3.77	0.008	1.02	0.81 – 1.28			0.865
Lumbar decompression	1.38	0.79 – 2.4	0.253	0.87	0.72 – 1.04			0.117
Lumbar arthrodesis	2.23	1.21 – 4.06	0.009	1.07	0.92 – 1.24			0.397

*OR* odds ratio, *CI* confidence interval.

## Discussion

Overall, our results confirmed that mortality following current orthopaedic surgery was low (0.11%). Although these results support previous reports [18], there was a marked difference in risk profile among patient subgroups. The multivariate analysis showed that postoperative mortality and morbidity were modestly associated with advanced age and strongly with the comorbidity burden as measured by the CCI. An increased CCI was the greatest risk factor for both in-hospital mortality and the occurrence of major complications. In addition, the use of lumbar arthrodesis and cervical laminoplasty were associated with increased risk of in-hospital mortality compared with TKA.

### Strengths and weaknesses of the study

This study is the largest (study population of 107,104) that analyzes the risks associated with current orthopaedic surgery in older adults. The DPC database is a large administrative database similar to National Inpatient Sample in the United States, and the data allows nationwide investigation and comparison of mortality and morbidity between stratified subgroups. In addition, the DPC database allows us to assess perioperative complications by differentiating comorbidities at admission and complications after admission.

In common with other studies using administrative data, a degree of misclassification or underreporting of outcome might have occurred. Although we could not verify data for each patient, we presume the level of miscoding is low because DPC data are coded by physicians and subject to an audit. Data are limited as the DPC database does not record some important clinical data, such as the severity of the disease, levels of the arthrodesis, or type of implant used. Despite these limitations, the results presented here provide important national estimates of inpatient morbidity and mortality after orthopaedic surgery.

### Comparison with other studies

The values for mortality for spine surgery (0.11%–0.20%) and primary TKA (0.066%) are similar to those reported in other large database studies [6-9,19-21]. In the present study, comorbidity burden (CCI ≥ 3) had the greatest impact both on in-hospital mortality and occurrence of major complications. In the National Surgical Quality Improvement Program, which examined outcomes for 3,475 patients undergoing spine surgery, identified age, and contaminated or infected wounds as independent predictors of mortality [10]. Pumberger et al. reported the highest odds for perioperative mortality for patients undergoing lumbar arthrodesis were among patients aged 75 years or older (OR, 4.35; reference: 45–46 years) and those with the comorbidities of congestive heart failure, coagulopathy, and liver disease [9]. Similarly, Memtsoudis et al. found that risk factors for mortality following TKA and THA included age (≥ 75 years; OR, 3.70), male gender, ethnicity, emergency admission, and comorbidity [8].

In these previous studies, each comorbid condition was analyzed separately. In the present study, we used the CCI because in elderly patients often present with multiple coexisting conditions. Our data demonstrate that presentation with coexisting conditions has a striking impact on postoperative adverse outcomes.

Risk profile also varies among surgical procedures. After adjusting for age, sex, and comorbidities, the risk for in-hospital death following lumbar arthrodesis or cervical laminoplasty was twice as high as the risk following TKA. There was no significant difference between lumbar decompression and TKA. Few previous studies have examined procedure-related risk differences among orthopaedic procedures. Memtsoudis et al. reported that TKA has a slightly lower risk of mortality than THA [8]. Deyo et al. demonstrated that lumbar arthrodesis, in particular complex fusion, compared with decompression alone, increased risk of 30-day mortality [5]. Although unadjusted factors may influence mortality rates, surgeons should be aware of the different risk profile among surgical procedures.

## Conclusions

Our findings suggest that an assessment of perioperative risks in elderly patients undergoing orthopaedic surgery should be stratified according to comorbidity burden and type of procedures, as well as by patient’s age. We believe that the findings of our study provide critical information for individual treatment recommendations for elderly patients.

## Abbreviations

DPC: Diagnosis procedure combination; TKA: Total knee arthroplasty; THA: Total hip arthroplasty; CCI: Charlson comorbidity index; OR: Odds ratios.

## Competing interests

The authors declare that they have no competing interests.

## Authors’ contributions

HC, HY, KT, and ST contributed to the conception and design of the study. HH, SS, ST, and KF contributed to the analysis, and all authors contributed to the interpretation. HC drafted the article; all authors revised it critically for important intellectual content and approved the final version submitted for publication. All authors read and approved the final manuscript.

## Pre-publication history

The pre-publication history for this paper can be accessed here:

http://www.biomedcentral.com/1471-2474/14/173/prepub
